# Acyclic artificial nucleic acid substitution expands the safe design space of gapmer ASOs by preventing P54nrb mislocalization

**DOI:** 10.1093/narmme/ugag036

**Published:** 2026-07-06

**Authors:** Jumpei Ariyoshi, Masaya Higuchi, Hiroyuki Oyama, Hiroyuki Asanuma, Yukiko Kamiya

**Affiliations:** Laboratory of Bioanalytical Chemistry, Kobe Pharmaceutical University, 4-19-1, Motoyamakita-machi, Higashinada-ku, Kobe 658-8558, Japan; Department of Biomolecular Engineering, Graduate School of Engineering, Nagoya University, Furo-cho, Chikusa-ku, Nagoya 464-8601, Japan; Laboratory of Bioanalytical Chemistry, Kobe Pharmaceutical University, 4-19-1, Motoyamakita-machi, Higashinada-ku, Kobe 658-8558, Japan; Department of Biomolecular Engineering, Graduate School of Engineering, Nagoya University, Furo-cho, Chikusa-ku, Nagoya 464-8601, Japan; Laboratory of Bioanalytical Chemistry, Kobe Pharmaceutical University, 4-19-1, Motoyamakita-machi, Higashinada-ku, Kobe 658-8558, Japan; Department of Biomolecular Engineering, Graduate School of Engineering, Nagoya University, Furo-cho, Chikusa-ku, Nagoya 464-8601, Japan

## Abstract

Gapmer-type antisense oligonucleotides (Gapmers) are promising therapeutic agents. However, their clinical potential is frequently limited by off-target toxicities. To address this issue, Gapmers have been optimized by modifying the ribose moiety or internucleotide linkage, but toxicity has not always been reduced. The toxicity is due to the unintended interactions between the ribose-type modified nucleic acids with phosphorothioate backbones and the endogenous proteins. We therefore hypothesized that the use of acyclic nucleic acids, which possess an entirely distinct structure to ribose, would solve the aforementioned issue. In this study, we demonstrate that the incorporation of an acyclic analog, serinol nucleic acid (SNA) or L-threoninol nucleic acid (L-*a*TNA), provides an alternative approach. Notably, substitution with SNA or L-*a*TNA effectively mitigated toxicity, even when conventional 2′-*O*-methyl modification was unsuccessful. This approach reduced cytotoxicity across multiple Gapmer sequences and designs in a position-dependent manner. Mechanistically, our investigation into these acyclic nucleic acids revealed that reduced toxicity was associated with suppression of P54nrb protein mislocalization. Furthermore, representative SNA- or L-*a*TNA-modified Gapmers exhibited markedly reduced hepatotoxicity *in vivo*. Collectively, these findings suggest that acyclic nucleic acids have potential as a useful chemical strategy for the development of safer Gapmer therapeutics.

## Graphical Abstract

**Graphical Abstract:**
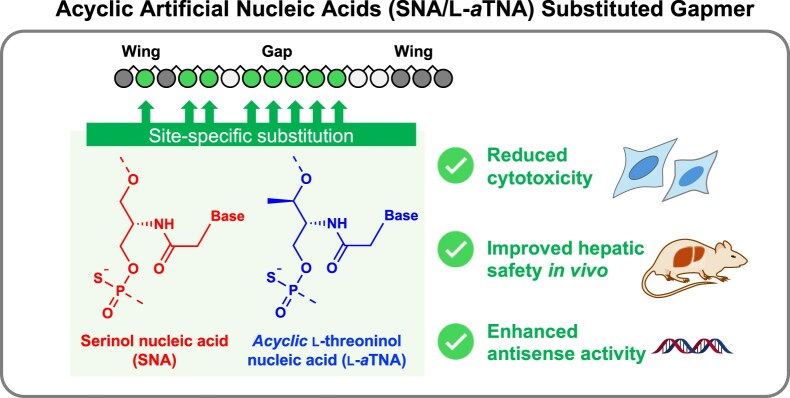

## Introduction

Nucleic acid therapeutics target specific RNAs and deliver therapeutic effects by inhibiting gene expression [1, 2]. In particular, Gapmer-type antisense oligonucleotides (Gapmers) can effectively regulate gene expression by degrading target RNA *via* RNase H [3, 4]. The design of Gapmers includes a central DNA “gap” region flanked by “wing” regions at the 5′ and 3′ ends. To increase nuclease resistance and binding affinity, these wings contain ribose-type artificial nucleic acids such as 2′-*O*-methoxyethyl (MOE) [5], locked nucleic acid (LNA) [6], and 2′-fluoro (2′-F) modifications [7]. Additionally, to enhance stability and improve pharmacokinetics, phosphorothioate (PS) backbones are incorporated throughout the oligonucleotide [8, 9]. The development of Gapmers with these chemical modifications is an active research topic. Although various Gapmers have been approved as therapeutic drugs [10–13], there are still unresolved safety concerns. Serious adverse effects, including hepatotoxicity, nephrotoxicity, or thrombocytopenia, have been reported during the drug development process [14–17].

There are two primary mechanisms of Gapmer toxicity. The first is hybridization-dependent off-target toxicity, in which the Gapmer hybridizes with nontarget mRNA or pre-mRNA owing to sequence similarity [18–20]. The risk of these off-target effects is particularly high in developmental candidates that use high-affinity modifications, such as LNA, as these can stabilize binding to mismatched RNAs [18, 21]. At the design stage, the use of *in silico* bioinformatic tools can avoid these sequence-dependent risks [19]. In addition, experimentally informed studies have shown that ASO toxicity is influenced by sequence motifs, secondary structure, and chemical modification patterns, highlighting the importance of integrating sequence- and chemistry-dependent considerations into Gapmer design [22, 23]. The second mechanism is hybridization-independent toxicity. This toxicity is increasingly recognized to result from unintended interactions between chemically modified ASO and proteins [24]. A combination of a phosphorothioate (PS) backbone and ribose-type artificial nucleic acids induces binding between Gapmers and numerous proteins, which is thought to be the origin of the interactions [25, 26]. Although the mechanisms of induction are not completely understood for many toxicities, the interaction between paraspeckle protein P54nrb and Gapmer is a well-known example of protein-mediated toxicity. The binding of a PS-modified Gapmer to P54nrb triggers the mislocalization of P54nrb from the paraspeckles to the nucleolus; this results in nucleolar stress and ultimately induces apoptosis [24, 27]. Consequently, a key strategy for the development of safer Gapmers is minimizing these undesirable protein-binding events through the careful placement of chemical modifications.

For example, the introduction of a single 2′-*O*-methyl (2′-OMe) modification at gap position 2 (G2-2′-OMe) was shown to significantly reduce protein binding and mitigate hepatotoxicity [27]. However, the effect of this modification appears to be context-dependent: in certain cases, the G2-2′-OMe modification was shown to paradoxically induce further protein binding [28, 29]. For certain Gapmers, the modification does not provide sufficient protection against hepatotoxicity [29]. Moreover, for Gapmers associated with late-onset neurotoxicity, the G2-2′-OMe modification may even exacerbate toxicity [30]. Thus, the effectiveness of this strategy is highly dependent on the nucleotide sequence. To develop more robust methods for mitigating toxicity, other chemical modification strategies have been explored. These include 5′-end modifications such as 5′-cyclopropylene (5′-CP) and 5′-methyl modifications, as well as nucleobase modification approaches [30–32]. Specifically, although 5′-CP has been primarily reported to mitigate toxicity when introduced at the G3 position, 5′-methyl modification is effective when introduced at the G3 and G4 positions. Although various modifications to the ribose scaffold have been explored, it is important to note that intrinsic toxicity often stems inherently from the ribose-based backbone. Despite the potential effectiveness of strategies that completely avoid ribose structures, alternative scaffolds that replace the ribose backbone remain largely unexplored.

Serinol nucleic acid (SNA) and acyclic L-threoninol nucleic acid (L-*a*TNA) are acyclic nucleic acid analogs synthesized from natural serine and L-threonine, respectively [33–35]. Despite substantial differences in their chemical structures to those of natural DNA and RNA, they exhibit strong RNA-binding affinity in a sequence-specific manner [36]. Owing to their unique chemical structures, they are not recognized by endogenous nucleases, which confers exceptional nuclease resistance [37]. Thus, they provide an attractive platform for oligonucleotide therapeutics, including exon-skipping ASOs, small-interfering RNAs, anti-miRNA oligonucleotides, and staple oligomers [37–42]. Importantly, we found significantly lower hepatotoxicity of a Gapmer with a wing region composed of SNA targeting *SGLT2* compared with a conventional 2′-MOE Gapmer [43]. Inspired by these findings, we reasoned that SNA and L-*a*TNA modifications could serve as a strategy to overcome the hepatotoxicity associated with conventional Gapmer ASOs. In this study, we investigated the potential of SNA and L-*a*TNA modifications for mitigating the intrinsic cytotoxicity of Gapmers. First, we demonstrated that SNA or L-*a*TNA substitutions result in a more significant reduction in cytotoxicity across multiple toxic Gapmer sequences. Through systematic positional screening, we identified specific modification patterns that can effectively separate antisense activity from cellular toxicity. Furthermore, this reduction in toxicity was mediated through the suppression of aberrant P54nrb mislocalization, which provides evidence to support the role of protein–oligonucleotide interactions. Finally, we validated the SNA/L-*a*TNA-modified Gapmers in an *in vivo* mouse model to achieve an improved safety profile.

## Materials and methods

### Oligonucleotide synthesis

Synthesis of all Gapmers used in this study was outsourced to Hokkaido System Science Co., Ltd. The complementary RNA oligonucleotide labeled with FAM at the 5′ end was purchased from Fasmac Co., Ltd.

### *T*_m_ measurements

To determine the melting temperature (*T*_m_), Gapmer and RNA were first mixed in a 1:1 molar ratio to a final concentration of 2 μM (duplex). The resulting duplexes were dissolved in buffer (10 mM sodium phosphate, pH 7.0, with 100 mM NaCl). The melting curves were obtained by measuring the change in absorbance at 260 nm versus temperature using a V-730 spectrophotometer (Jasco Co., Ltd.). Thermal denaturation temperatures (*T*_m_) were calculated as the first derivative of the melting curve using the instrument’s integrated software.

### Cell culture

Human cervical cancer cells (HeLa) and mouse embryonic fibroblast cells (3T3-L1) were maintained in Dulbecco’s modified Eagle’s medium (DMEM) supplemented with 10% fetal bovine serum (FBS) and 1% penicillin–streptomycin. Cell culture was performed at 37°C in a humidified atmosphere containing 5% CO_2_.

### MTS assay

HeLa or 3T3-L1 cells were seeded into 96-well plates at a density of 20,000 cells/well and cultured for 24 h. After culture, cells were then transfected with Gapmer using Lipofectamine 2000 (Invitrogen) in Opti-MEM (Thermo Fisher Scientific) in accordance with the manufacturer’s protocol. After a 6-h transfection period, the medium was replaced with fresh DMEM containing 10% FBS and antibiotics. At 24 h post-transfection, cell viability was measured using the CellTiter 96^®^ AQueous One Solution Cell Proliferation Assay kit (Promega) and a BioTek Synergy HTX microplate reader (Agilent) in accordance with the manufacturer’s instructions.

### Quantitative real-time PCR (RT-qPCR)

3T3-L1 cells were seeded in a 24-well plate (IWAKI) at a density of 30,000 cells/well and cultured for 24 h. The cells were then transfected with Gapmer using Lipofectamine 2000 (Invitrogen) as described above in the “MTS assay” section. After 8 h, the medium was replaced with fresh culture medium. At 24 h after the start of transfection, the cells were collected, and total RNA was subsequently isolated using the RNeasy Mini Kit (Qiagen) in accordance with the manufacturer’s protocol. cDNA was synthesized from the total RNA using ReverTra Ace^®^ qPCR RT Master Mix with gDNA Remover (TOYOBO). Quantitative real-time PCR was performed using KAPA SYBR^®^ FAST (Roche) and a LightCycler^®^ 96 (Roche). The mRNA level was normalized to that of GAPDH. Primers with the following sequences were used: *CXCL12* forward, 5′-CCAGAGCCAACGTCAAGCAT-3′; reverse, 5′-CAGCCGTGCAACAATCTGAA-3′; *GAPDH* forward, 5′-CTCCCACTCTTCCACCTTCG-3′; reverse, 5′-GCCTCTCTTGCTCAGTGTCC-3′.

### Immunofluorescence staining

Cultured HeLa cells (20,000 cells/well) were plated onto glass-bottomed chambers and transfected with Gapmers using Lipofectamine 2000 (Invitrogen). After a 4-h incubation period, the cells were washed with PBS, fixed with 4% paraformaldehyde phosphate buffer solution for 30 min, and washed again with PBS. The cells were then permeabilized with permeabilization buffer (1× PBS, 10 mM glycine, 0.2% Triton X-100) for 60 min, washed with PBS, and blocked by incubation in 3% BSA in PBST (phosphate-buffered saline containing 0.05% Tween 20) for 1 h at room temperature. Subsequently, the cells were incubated with a primary antibody against P54nrb (sc-376865, 1:125 dilution, Santa Cruz Biotechnology) and/or an antibody against NCL (ab22758, 1/1000, Abcam) at 4°C for 24 h. The chambers were washed with PBST and the cells were incubated with Alexa Fluor 488-conjugated mouse anti-mouse IgG secondary antibody (A1101, 1:1000 dilution, Invitrogen), and DAPI for 1 h at room temperature. Fluorescence imaging was performed using an FV3000 confocal laser scanning microscope (Olympus). The nucleolar mislocalization of P54nrb was scored through P54nrb enrichment in nucleoli (nucleolin-positive when stained; otherwise, this was identified based on DAPI exclusion and nuclear morphology). Cells with ≥1 positive nucleolus were counted, with >100 cells per condition analyzed.

### RNase H1 cleavage assay

Recombinant human RNase H1 (RNASEH1; Wuhan Huamei Biotech Co., Ltd., formerly Cusabio LLC) was used for *in vitro* analysis of cleavage. A 5′-FAM-labeled RNA target (2 µM) was hybridized with each Gapmer-ASO (1 µM) in assay buffer (10 mM Tris–HCl pH 7.8, 40 mM KCl, 8 mM MgCl₂, and 1 mM DTT). The duplexes were incubated with RNase H1 (0.3 µM; final volume 10 µl) at 37°C for 20 min. Samples were frozen in liquid nitrogen to stop the reaction and were then stored at −80°C until analysis. Prior to electrophoresis, samples were mixed with equal volumes of loading buffer (95% formamide, 10 mM EDTA). Samples were separated by 20% denaturing PAGE (8 M urea) at 300 V for 120 min.

Fluorescent RNA fragments were visualized using an ImageQuant LAS 4000 imager (Cytiva) with Epi-B Set (LAS) illumination (460 nm excitation/515 nm long-pass emission filter). The band intensities were quantified using ImageQuant TL software, and cleavage efficiency (%) was calculated using the following equation:


\begin{eqnarray*}
100 - \left( {\frac{{\textit{intensity}{\mathrm{\ }}of{\mathrm{\ }}\textit{full}{\mathrm{\ }}\textit{length}{\mathrm{\ }}RNA{\mathrm{\ }}\textit{band}}}{{\textit{total}{\mathrm{\ }}\textit{lane}{\mathrm{\ }}\textit{intensity}}}} \right) \times 100.
\end{eqnarray*}

### Animal study

Animal experiments were performed in accordance with the ethical guidelines for Kobe Pharmaceutical University Committee for Animal Care and Use (approval no. 2025-017). Five-week-old male C57BL/6J mice were purchased from Japan SLC, Inc. (Shizuoka, Japan) and housed under specific-pathogen-free conditions with free access to food and water. Oligonucleotides were dissolved in sterile phosphate-buffered saline. Mice were administered a single intravenous (*i.v*.) injection of PBS (mock), nontoxic control Gapmer, parental Gapmer (Gap-*Cxcl12*), or SNA/L-*a*TNA-substituted Gapmers at doses of 10 mg/kg (*n* = 4 per group). Blood samples were collected at 96 h postinjection. After serum was isolated by centrifugation, the levels of aspartate aminotransferase (AST), alanine aminotransferase (ALT), and lactate dehydrogenase (LDH) were measured by Oriental Yeast Co., Ltd. (Tokyo, Japan).

## Results

### Substitution with SNA or L-*a*TNA effectively reduces cytotoxicity in a position-dependent manner

**Figure 1.**
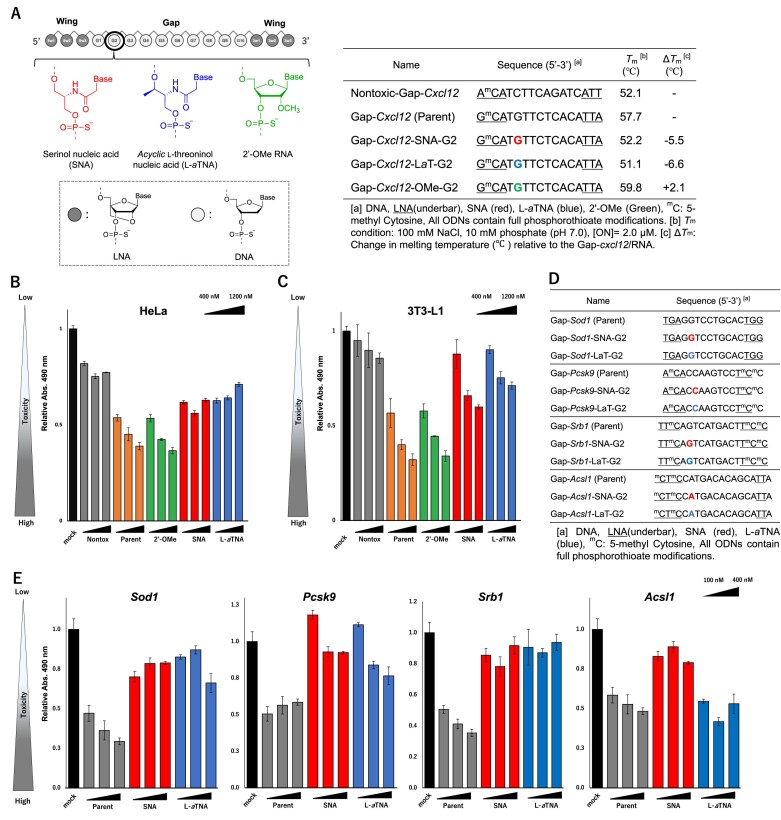
Substitution with acyclic nucleic acids at the G2 position of Gapmer reduces cytotoxicity. (**A**) The chemical structure of Gap-*Cxcl12* composed of LNA and DNA with phosphorothioate modifications. The sequences of modified Gap-*Cxcl12* with a SNA, L-*a*TNA, or 2′-OMe substitution at the G2 position. Nontoxic Gapmer was used as a control with different sequences that target *Cxcl12* and do not have cytotoxic effects. Assessment of the cytotoxicity of modified Gap-*Cxcl12s* in HeLa cells (**B**) and 3T3-1 cells (**C**) using the MTS assay. Cells were transfected with Gapmer (400–1200 nM) using Lipofectamine 2000 and cell viability was measured 24 h after transfection using an MTS assay. The data are presented as mean ± SD (*n* = 3). (**D**) Sequence of Gapmers targeting Sod1, *Pcsk9, Srb1*, and *Acsl1* and the substitution of the G2 position with either SNA or L-*a*TNA. (**E**) Evaluation of the cytotoxicity of the modified Gapmers in HeLa cells using the MTS assay. The data are presented as mean ± SD (*n* = 3).

A previous report established that substitution of the DNA nucleotide at gap position 2 (G2) with a 2′-OMe backbone can mitigate Gapmer cytotoxicity [27]. First, we investigated the effect of substitution at the G2 position with either SNA or L-*a*TNA on the cytotoxicity of Gapmers. To confirm this, we used previously reported data. The highly cytotoxic 3-10-3 LNA Gapmer targeted to *Cxcl12* mRNA (Gap-*Cxcl12*) is composed of LNA at the wing region and is fully phosphorothioated. The high toxicity of Gap-*Cxcl12* was confirmed using the MTS assay. We designed Gapmers by introducing a single 2′-OMe, SNA, or L-*a*TNA substitution into the G2 position of the Gap-*Cxcl12* sequence (Fig. 1A). The MTS assay showed that the viability of cells transfected with the 2′-OMe substituted Gap-*Cxcl12* was equivalent to that of cells transfected with Gap-*Cxcl12* (Fig. 1B). In contrast to previous reports, we found that the substitution of 2′-OMe at the G2 position did not reduce the cytotoxicity of Gap-*Cxcl12 *[27]. In contrast, cells transfected with SNA or L-*a*TNA-substituted Gap-*Cxcl12* exhibited good viability (Fig. 1B). This finding indicates that substitution with SNA or L-*a*TNA at the G2 position reduces the cytotoxicity of Gap-*Cxcl12*. The effect of substitution of SNA or L-*a*TNA on the cytotoxicity of Gap-*Cxcl12* was also examined in 3T3-L1 cells. Similar to the effects in HeLa cells, substitution of 2′-OMe at the G2 position of Gap-*Cxcl12* did not reduce cytotoxicity; however, substitution of SNA or L-*a*TNA did reduce toxicity (Fig. 1C). Therefore, the reduction in cytotoxicity achieved by substitution of SNA or L-*a*TNA at the G2 position was applicable to different types of cell lines. Furthermore, we assessed the effects of SNA or L-*a*TNA substitution on the cytotoxicity of Gapmers with different nucleobase sequences and lengths. We evaluated the effect of SNA or L-*a*TNA substitution at the G2 position on the cytotoxicity of four previously reported cytotoxic Gapmers: the 3-10-3 LNA Gapmer *Sod1*, the 3-8-3 LNA Gapmer *Pcsk9*, the 3-10-3 LNA Gapmer *Srb1*, and the 3-12-2–1 LNA Gapmer *Acsl1* (Fig. 1D). As shown in Fig. 1E, the substitution of either SNA or L-*a*TNA at the G2 position effectively reduced the toxicity of Gap-*Sod1*, Gap-*Pcsk9*, and Gap-*Srb1* in HeLa cells. For Gap-*Acsl1*, the substitution of SNA reduced toxicity, whereas L-*a*TNA substitution did not. These trends were largely reproduced in 3T3-L1 cells (Supplementary Fig. S1). Collectively, these results demonstrate that G2 substitution with acyclic nucleic acids can reduce Gapmer cytotoxicity across multiple sequences and in both HeLa and 3T3-L1 cells. These findings indicate that acyclic nucleic acid substitution represents broadly useful strategy for mitigating Gapmer cytotoxicity, while also showing that the extent of the toxicity reduction depends on the target sequence and the specific acyclic scaffold utilized. Importantly, SNA or L-*a*TNA substitution reduced cytotoxicity even in the *Cxcl12*-targeting gapmer sequence, where 2′-OMe substitution at G2 was insufficient. This suggests that acyclic nucleic acid offers a highly potent chemical strategy that overcomes the limitations of conventional ribose-based modification.

**Figure 2.**
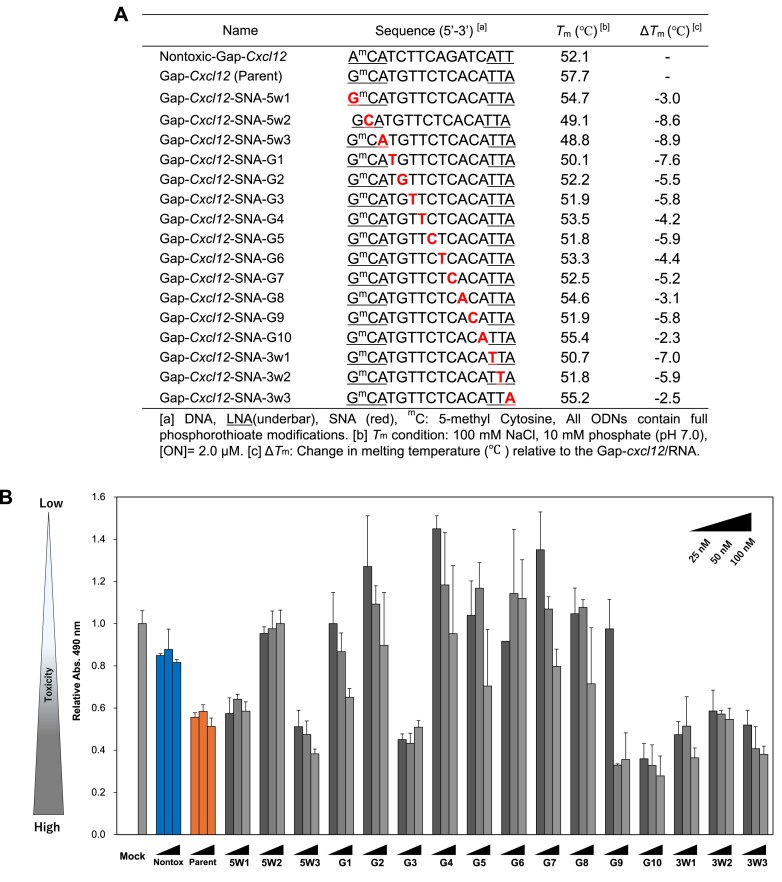
Positional effects of the substitution of SNA on Gapmer cytotoxicity. (**A**) Sequences of the parental Gapmer (Gap-*Cxcl12*) and its SNA-substituted Gapmers (e.g. Gap-*Cxcl12*-SNA-G2) and melting temperature (*T*_m_) of the Gapmer/complementary RNA duplex. LNA-modified nucleotides are underlined; SNA is shown in red. (**B**) The cytotoxicity of HeLa cells treated with each Gap-*Cxcl12* bearing a single SNA substitution at each position, as determined using the MTS assay. Data are shown as mean ± SD (*n* = 3).

After confirming the efficacy of substitution at the G2 position for reducing cytotoxicity, we investigated whether the modification of other Gapmer positions with SNA could also reduce cytotoxicity. Consequently, a systematic positional scan was conducted in which each site was substituted with SNA in the entire toxic Gap-*Cxcl12* sequence (Fig. 2A). To evaluate the impact of these substitutions on duplex stability, we measured the melting temperatures (*T*_m_) of the parent Gap-*Cxcl12*/RNA duplex and all SNA- or L-*a*TNA-substituted Gapmer/RNA duplexes. Compared with the parent duplex (*T*_m_ = 57.7°C), the *T*_m_ values of all SNA- or L-*a*TNA-substituted duplexes were consistently decreased. The results of the MTS assay in HeLa cells demonstrated a clear position-dependent effect on the extent of cytotoxicity (Fig. 2B). A significant reduction in toxicity was observed not only at the G2 position but also at several other sites. Notably, substitution at the 5′-wing (5w2) and within the 5′ half of the gap region (positions G1–G8, excluding G3) proved effective. In contrast, modifications in the 3′-wing region did not reduce cytotoxicity. This position-dependent toxicity reduction by the single SNA substitution was also reproduced in 3T3-L1 cells (Supplementary Fig. S2). A similar trend was observed for L-*a*TNA substitution (Supplementary Fig. S3). Collectively, these positional scan results successfully defined the specific regions where the substitution of SNA or L-*a*TNA can effectively mitigate Gapmer cytotoxicity. The observation that the reduction in toxicity is highly dependent on the substitution site strongly suggests that the SNA or L-*a*TNA modification specifically inhibits a critical interaction within the mechanism of Gapmer-induced toxicity.

### SNA and L-*a*TNA substitution reduces P54nrb mislocalization

To investigate the underlying mechanism of the potent reduction in cytotoxicity achieved by our acyclic analogs, we considered the mislocalization of the paraspeckle protein P54nrb, which has been identified as a key protein that interacts with cytotoxic Gapmers. The resultant complex is mislocalized from the nucleoplasm to the nucleolus *via* RNase H, and this process subsequently induces nucleolus stress and apoptosis [27]. Because of this mechanism, we hypothesized that SNA and L-*a*TNA reduce cytotoxicity by preventing P54nrb mislocalization, potentially by altering the geometry and conformational dynamics of the backbone, and consequently attenuating nonspecific protein interactions that drive P54nrb mislocalization.

**Figure 3.**
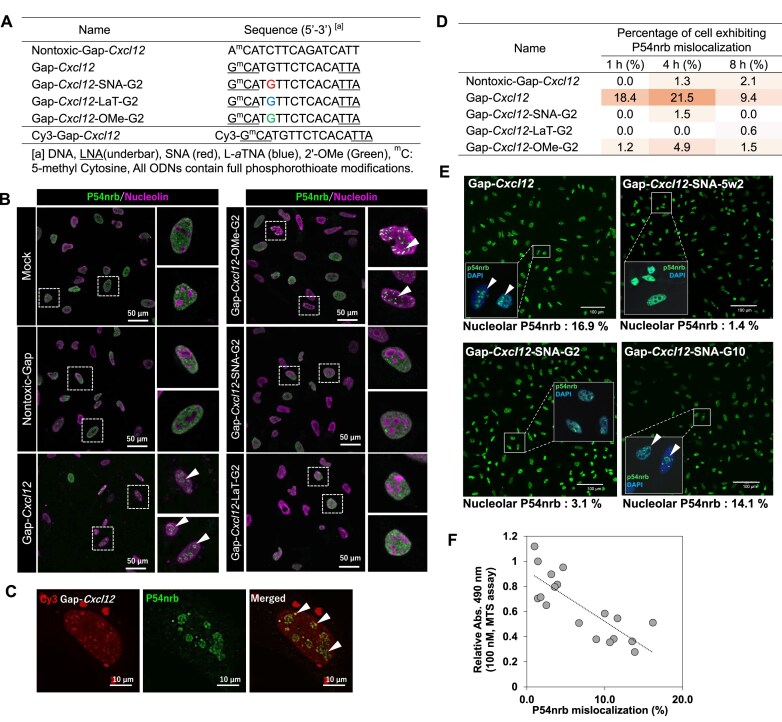
The localization of P54nrb in cells transfected with SNA or L-*a*TNA-substituted Gap-*Cxcl12*. (**A**) Sequences of Gapmers substituted with SNA, L*-a*TNA, or 2′-OMe at position G2. (**B**) Immunofluorescence staining of P54nrb (green) and nucleolin (magenta) in HeLa cells at 4 h after transfection with 1,200 nM Gapmer. White arrows indicate the mislocalization of P54nrb to the nucleolus. Scale bar, 50 μm. (**C**) Representative images of Cy3-labeled toxic Gap-*Cxcl12* (red), P54nrb (green), and nucleolin (magenta) in HeLa cells at 4 h after transfection. Scale bar, 10 μm. (**D**) Percentages of cells exhibiting P54nrb localization in the nucleolus. HeLa cells were transfected for 1, 4, and 8 h with 1,200 nM Gapmer, and >100 cells per condition were scored in a single representative experiment. (**E**) Representative immunofluorescence images of P54nrb (green) and nucleolin (magenta) in HeLa cells treated with the indicated SNA-substituted Gap-*Cxcl12*. White arrows indicate the mislocalization of P54nrb in the nucleoli. The percentage of cells exhibiting nucleolar-localized P54nrb is presented below each image, and >200 cells per condition were scored. (**F**) Correlation between Gapmer cytotoxicity and P54nrb mislocalization. The cell viability (determined by absorbance at 490 nm derived from the MTS assay; Fig. 2B) is plotted against the percentage of cells exhibiting nucleolar P54nrb mislocalization.

First, we evaluated the impact of SNA/L-*a*TNA substitution of Gap-*Cxcl12* at position G2 (Gap-*Cxcl12*-SNA-G2 or Gap-*Cxcl12*-LaT-G2) on the mislocalization of P54nrb using immunofluorescence imaging analysis in HeLa cells. P54nrb was correctly localized in the nucleoplasm of cells treated with nontoxic Gapmer (Fig. 3B). As previously reported, the mislocalization of P54nrb to the nucleolus was observed in cells transfected with the toxic Gap-*Cxcl12* (indicated by white arrows in Fig. 3B). In contrast, a marked decrease in P54nrb mislocalization was evident in cells treated with Gapmer, in which SNA or L-*a*TNA substitutions were introduced at the G2 position (Fig. 3B). It was also observed that the 2′-OMe modification was less effective in suppressing P54nrb mislocalization than substitution of SNA and L-*a*TNA (Fig. 3B). The colocalization of Cy3-labeled toxic Gap-*Cxcl12* was observed in the nucleus (Fig. 3C). The rate of mislocalization of P54nrb was then quantified (Fig. 3D). The highest rate of P54nrb mislocalization (21.5%) in the parent toxic Gapmer-treated cells was at 4 h after transfection, whereas cells treated with Gap-*Cxcl12* substituted with SNA at the G2 position exhibited a markedly low rate of P54nrb mislocalization (1.5% for Gap-*Cxcl12*-SNA-G2 and 0.0% for Gap-*Cxcl12*-LaT-G2) (Fig. 3D). The rate of mislocalization in cells treated with 2′-OMe-substituted Gapmer was moderate (4.9%).

Furthermore, we evaluated the effect of SNA substitution at other positions on P54nrb localization in HeLa cells (Fig. 3E and Supplementary Fig. S2) and found that P54nrb localization was normal in cells that were transfected with SNA-substituted Gap-*Cxcl12*s at 5w2, G2, or G5 (low P54nrb mislocalization rates of 1.4%, 3.1%, and 1.4%, respectively). Hence, the cytotoxicity of this Gapmer was relatively low. In contrast, the Gapmer substituted with SNA at position G10 did not reduce cytotoxicity to the same extent. Instead, it promoted the mislocalization of P54nrb in the cells, and the rate of mislocalization remained high (14.1%). To summarize the findings across all SNA-substituted Gapmers, a plot of cytotoxicity versus P54nrb mislocalization rate was generated (Fig. 3F and Supplementary Fig. S4). The resulting plot shows a distinct downward trend, in which a higher degree of protein mislocalization is consistently associated with a reduction in cell survival. This strong correlation provides support for the hypothesis that the cytotoxicity of Gapmers is driven by aberrant interaction and the subsequent mislocalization of P54nrb. Furthermore, SNA- and L-*a*TNA-substituted Gap-*Sod1* suppressed P54nrb mislocalization in 3T3-L1 cells, supporting that this effect is not limited to the HeLa/Gap-*Cxcl12* system (Supplementary Fig. S5). Together, these results strongly support a close association between SNA/L-*a*TNA-mediated cytotoxicity reduction and suppression of P54nrb mislocalization.

### SNA and L-*a*TNA substitutions modulate the antisense activity of Gapmers

**Figure 4.**
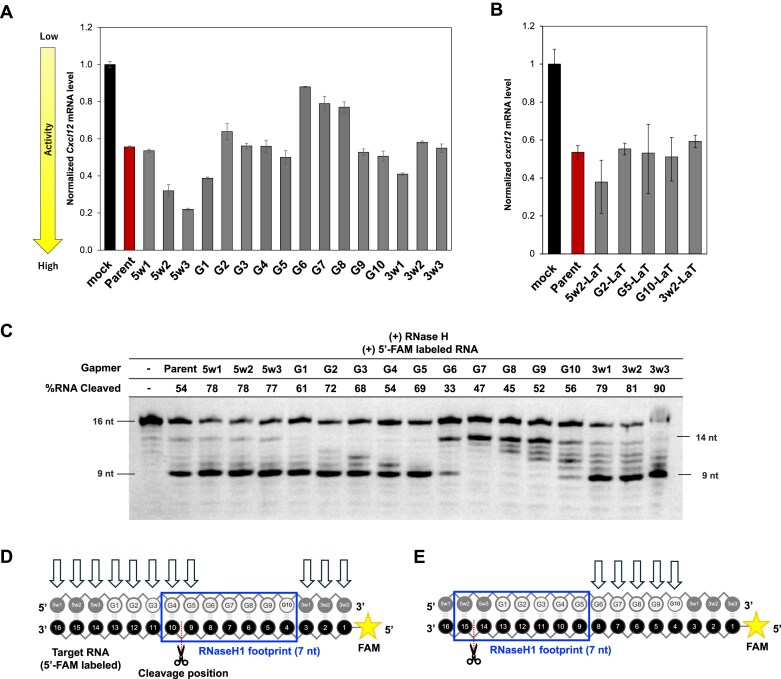
**A** and **B**) Antisense activity of the SNA- or L-*a*TNA-substituted Gap-*Cxcl12* and *Cxcl12* mRNA expression in 3T3-L1 cells were analyzed using RT-qPCR at 24 h after transfection of each Gapmer (100 nM). The bar graph shows the mean ± SD of normalized *Cxcl12* mRNA expression from *n* = 3 replicates. (**C**) Denaturing PAGE analysis of the RNA products cleaved by hRNaseH1 in the presence of the indicated SNA-substituted Gapmers. Above each gel lane, the percentage of cleaved RNA is presented. The gel image and quantification were obtained from a representative experiment. (**D, E**) Schematics of the Gapmer/RNA duplex showing the RNase H1 footprint and inferred cleavage position. The inferred RNase H1 footprint was assigned based on the reported structure of human RNase H1 in complex with a DNA/RNA hybrid [47]. White arrows indicate the SNA substitution sites: substitutions outside G6–G10 predominantly produced a 9-nt fragment (D), and substitutions at G6–G10 shifted the predominant product to 14 nt (E).

Next, we examined the antisense activity of SNA-substituted gapmers. The activities of the SNA-substituted Gapmers were analyzed by measuring *Cxcl12* mRNA levels (Fig. 4A). Compared with the antisense activity of the parent toxic Gap-*Cxcl12*, Gap-*Cxcl12* with a single SNA substitution in the central gap region, particularly at positions G6, G7, or G8, exhibited reduced antisense activity. The antisense activities of Gap-*Cxcl12*s substituted with SNA at 5w1, G2-G5, G9-G10, or 3w2-3w3 were comparable with those of the parent Gapmer. Interestingly, the incorporation of SNA substitution at 5w2, 5w3, G1, or 3w1 enhanced antisense activity compared with the parent sequence. The antisense activities of L-*a*TNA-substituted Gapmers were also analyzed, and a similar position-dependent trend was observed (Fig. 4B). As shown in Fig. 2A, SNA or L-*a*TNA substitution generally decreased the *T*_m_ values, with relatively large decreases observed for the Gapmers modified at 5w2, 5w3, G1, or 3w1. These data suggest that moderate destabilization of the Gapmer/RNA duplex at these positions may facilitate the dissociation of the cleaved RNA product, thereby promoting RNase H turnover and enhancing overall antisense activity.

To further characterize positional effects, we assessed the RNase H-mediated RNA cleavage efficiency of RNA/modified Gapmer duplexes using an *in vitro* cleavage assay (Fig. 4C). For this assay, 5′-FAM-labeled target RNA was used. The parent Gapmer-mediated cleavage of the target RNA by RNaseH occurred with a 9-nucleotide fragment detected as the major product. This length is consistent with the major fragments generally reported for 3-10-3 Gapmers [44–46]. Other than those modified with G6, G7, G8, G9, or G10, most SNA-substituted Gapmers yielded the same 9-nucleotide RNA fragment as their parent Gapmer. Interestingly, RNA cleavage efficiencies mediated by these SNA-modified Gapmers were higher compared with that of the non-SNA-modified Gap-*Cxcl12*. In contrast, substitutions at the G6, G7, G8, G9, or G10 positions shifted the major product to a distinct 14-nucleotide fragment and reduced the overall cleavage efficiency (Fig. 4C). The schematics presented in Fig. 4D and E provide a comprehensive representation of the inferred RNase H1 footprint on the Gapmer/RNA duplex, along with the corresponding cleavage register that results in the predominant 9-nt (Fig. 4D) or 14-nt (Fig. 4E) RNA fragments [47]. Collectively, these results demonstrate that although RNase H-mediated cleavage is retained for most SNA-substituted Gapmers, both the cleavage efficiency and the resulting product profile are highly position-dependent.

### SNA and L-*a*TNA substitution mitigates Gapmer-induced hepatotoxicity *in vivo*

**Figure 5.**
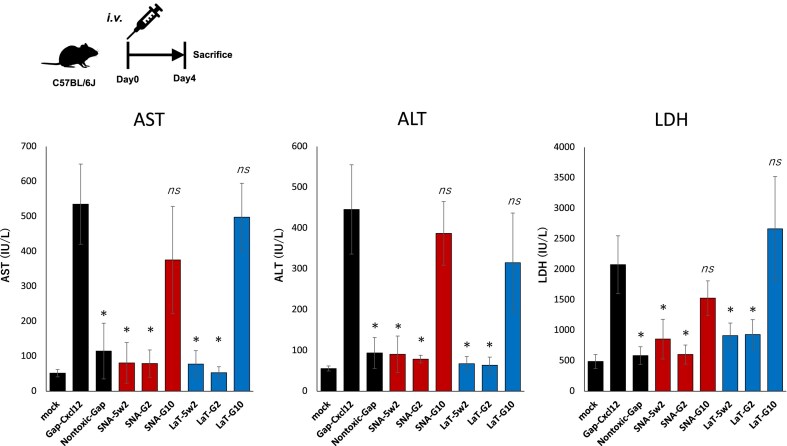
Substitution of SNA or L-*a*TNA effectively mitigates Gapmer-induced hepatotoxicity *in vivo*. Male C57BL/6J mice (5 weeks old) were administered a single intravenous (*i.v*.) injection of PBS (mock), nontoxic Gap-*Cxcl12*, Gap-*Cxcl12*, or SNA- or L-*a*TNA-substituted Gap-*Cxcl12* at positions G2, G10, or 5w2 (Gap-*Cxcl12*-SNA-G2, -G10, -5w2, and Gap-*Cxcl12*-LaT-G2, -G10, -5w2) at doses of 10 mg/kg. Blood samples were collected at 96 h postinjection for biochemical analysis. Data are expressed as the mean ± SD (*n* = 4 per group), and statistical significance was determined using Welch’s t-test with Holm correction for multiple comparisons (each group versus Gap-*Cxcl12*). *P < .05.

To build on the *in vitro* findings, we evaluated the *in vivo* hepatotoxicity of Gap-*Cxcl12* and its SNA- or L-*a*TNA-substituted Gapmers. Therefore, we selected three representative substitution sites (G2, G10, and 5w2) and examined whether these substitutions mitigated liver injury in mice. C57BL/6J mice (*n* = 4 per group) were intravenously injected with Gap-*Cxcl12* (a nontoxic Gap-*Cxcl12* control) or substituted Gapmers (Gap-*Cxcl12*-SNA-G2, Gap-*Cxcl12*-SNA-G10, Gap-*Cxcl12*-SNA-5w2, and the corresponding L-*a*TNA-substituted Gapmers). Liver injuries were assessed using serum ALT and AST, which are widely regarded as canonical indicators of hepatocellular injury, and LDH, which is a general marker of cellular/tissue damage (Fig. 5). Additional serum parameters are presented in Supplementary Fig. S6. Gap-*Cxcl12* induced marked elevations of AST, ALT, and LDH relative to the nontoxic Gap-*Cxcl12* control, which were consistent with liver injury and accompanying tissue damage. In contrast, substitution of SNA or L-*a*TNA at G2 or 5w2 significantly suppressed the elevation of AST, ALT, and LDH, with levels of these markers close to those in the nontoxic control. Conversely, substitution at G10 with SNA or L-*a*TNA provided no apparent mitigation, with AST, ALT, and LDH levels similar to those observed with the toxic parental Gap-*Cxcl12*. Overall, these results demonstrate that the introduction of SNA or L-*a*TNA at selected positions can mitigate Gapmer-induced hepatotoxicity *in vivo*; however, the degree of protection depends on the substitution site.

## Discussion

Chemical modifications are critical for developing antisense oligonucleotides into viable therapeutics. Although the combination of a PS backbone and ribose-based analogs has become the standard platform for Gapmer design, it is also recognized as a primary culprit of hybridization-independent toxicities, with hepatotoxicity being a major and well-documented concern [22]. These toxicities have been reported to arise from unintended protein interactions. Consequently, the replacement of specific nucleotides with alternative chemistries able to disrupt undesirable protein interactions has become a practical strategy. In the present study, we demonstrated that the incorporation of SNA or L-*a*TNA into Gapmers significantly alleviated toxicity without compromising antisense activities. This result suggests that modification with these acyclic analogs is a versatile means to enhance the therapeutic index of Gapmer-based antisense oligonucleotides.

Based on our assays using HeLa and 3T3-L1 cells, the substitution of 2′-OMe at the G2 position of Gapmers did not effectively mitigate cytotoxicity (Fig. 1A). However, the substitution of acyclic artificial nucleic acids (SNA and L-*a*TNA) at the G2 position markedly improved cell viability (Fig. 1B and C). This clear difference in outcome between G2 substitution with 2′-OMe and SNA/L-*a*TNA suggests that the structural differences between the acyclic scaffold and the ribose scaffold contribute to the superior mitigation of cytotoxicity. The robustness of G2 substitution with acyclic analogs (SNA/L-*a*TNA) was demonstrated through its consistent efficacy across Gapmers of varying sequences and lengths, including the 3-10-3 LNA Gapmer for *Sod1* and the 3-8-3 LNA Gapmer for *Pcsk9* (Fig. 1D). These results indicate that for Gapmers with a gap length of ~10 nucleotides, toxicity mitigation *via* SNA or L-*a*TNA substitution is a highly versatile approach. Interestingly, for Gapmers targeting *Acsl1*, the SNA substitution retained efficacy, but efficacy was reduced with L-*a*TNA (Fig. 1D). This suggests that as the gap length increases, differences in the chemical structure or helicity of the SNA and L-*a*TNA backbones start to exert an influence on their relative ability to reduce toxicity. This sequence- and length-dependent effect implies that the protective effect may be governed by subtle structural adaptations or local sequence contexts.

Further, we investigated the positional dependency of toxicity mitigation through the systematic substituting of SNA into each position in the Gapmer. Our analysis revealed that the protective effect of SNA was not confined to a single site (Fig. 2B). Specifically, the toxicity was mitigated across a broad 5′-side window, including the 5′ wing (e.g. 5w2) and multiple positions within the DNA gap, ranging from the 5′ portion toward the central region (specifically G1 and positions throughout the gap, excluding G3). This extensive and effective range is a clear contrast with previously described modifications. In previous studies, 2′-OMe mainly reduced toxicity when substituted at G2, with only minimal effects observed at other sites [27]. Similarly, for 5′-modifications, such as 5′-methyl or 5′-CP, the effective range was primarily confined to the third and fourth positions of the DNA gap [30, 31]. A combination of 5′-CP at the third position with a phosphodiester (PO) linkage has been reported as a highly effective strategy for toxicity reduction [30]. Another relevant precedent is found in methoxypropylphosphonate ASOs (MOP-ASOs), in which selected PS linkages are replaced with charge-neutral alkyl phosphonate linkages [48]. In that study, toxicity was most effectively reduced by MOP incorporation at gap positions 2 or 3 from the 5′ side, and this effect was associated with suppression of P54nrb nucleolar mislocalization. In comparison, SNA substitution conferred protection across a broader positional window than these previously reported position-restricted strategies, extending from the 5′ wing to multiple positions within the gap region. Thus, SNA and L-*a*TNA substitutions provide a complementary strategy to MOP-type linkage modification by targeting the nucleoside scaffold itself, thereby expanding the chemical repertoire for reducing hybridization-independent toxicity of gapmer ASOs. The robust and spatially extensive efficacy of SNA substitution likely stems from the fundamental divergence of its structure from those of ribose-based scaffolds. Chemical modifications to ribose-type nucleic acids are known to introduce bulkiness and differences into the rotational preference of the ribose structure. Although these modifications have the potential to suppress protein binding by altering the local environment, they still operate within the constraints of a furanose geometry. In contrast, SNA possesses an entirely different acyclic chemical backbone. Not only does the acyclic nature of SNA disrupt local protein–DNA interactions more significantly, it also induces more profound alterations in the overall duplex helicity. We propose that this superior ability to inhibit nonspecific protein binding, irrespective of the specific sequence context, allows SNA to mitigate cytotoxicity across a much wider range of positions than conventional ribose-type modifications.

The *in vivo* safety data of acyclic nucleic acid-modified Gapmers are consistent with the positional effects observed *in vitro* (Fig. 5 and Supplementary Fig. S3). Specifically, the introduction of SNA or L-*a*TNA at G2 or 5w2 substantially attenuated the Gap-*Cxcl12*-induced elevation of serum markers of hepatocellular injury (AST and ALT) as well as the general tissue-injury marker LDH. Conversely, substitution at G10 resulted in minimal to no improvement, with marker levels remaining similar to the parental toxic Gapmer. However, serum parameters reflecting broader systemic and renal effects (TP, UN, CRE, and ALP) were unchanged, and no evidence of nephrotoxicity was observed with either the parental Gapmer or any substituted Gapmer (Supplementary Fig. S3). Together, these results indicate that the position-optimized incorporation of acyclic nucleic acids is a robust strategy for the mitigation of Gapmer-associated hepatotoxicity *in vivo*.

Our systematic positional scan provided critical mechanistic insights into the reduction in toxicity of Gapmers following SNA or L-*a*TNA substitution. A strong quantitative correlation was observed between the reduction in cytotoxicity and the suppression of P54nrb mislocalization across various substitution positions. Among the SNA-substituted Gapmers, positions that effectively reduced cytotoxicity, such as 5w2, and multiple positions on the 5′ half of the gap from G1 to G8 (with the exception of G3) closely matched the positions where P54nrb mislocalization was minimized (Fig. 3F). Specifically, SNA or L-*a*TNA substitution at the G2 position exhibited substantially more pronounced suppression of P54nrb mislocalization compared with 2′-OMe substitution at the G2 position (Fig. 3D). As P54nrb mislocalization is triggered by unintended binding to the Gapmer, these results suggest that acyclic analogs reduce this protein interaction. Furthermore, the observation that SNA substitutions at Gap positions other than G2 also resulted in an accompanying reduction in both toxicity and P54nrb mislocalization suggests that these specific Gap positions are also directly involved in the interaction with toxicity-inducing proteins. Collectively, the results of this study provide substantial evidence that the toxicity-reducing effect of SNA and L-*a*TNA occurs through the prevention of P54nrb mislocalization. This evidence substantiates the notion that SNA and L-*a*TNA substitution mitigates binding to P54nrb, which is a key step in hybridization-independent toxicity. Given that PS-ASO-associated toxicity is linked to a broader network of ASO-binding proteins, including RNA-binding and paraspeckle proteins such as SFPQ and PSPC1, [28, 49] future studies are needed to determine whether SNA and L-*a*TNA substitutions also suppress the abnormal interaction or mislocalization of these factors. Although the precise interaction mechanisms remain to be fully elucidated through structural or binding assays, these results provide a strong molecular basis for the enhanced safety profile of acyclic-modified Gapmers.

With regard to antisense activity, our results suggest that the effect of SNA and L-*a*TNA substitution on antisense activity is governed by two positional factors: (i) the thermodynamic stability (*T*_m_) of the duplex formed with the target RNA; and (ii) the structural compatibility of the duplex of modified Gapmer/RNA with RNase H. A single substitution with the acyclic analog results in a moderate reduction in duplex stability (*T*_m_) of the Gapmer and target RNA (Fig. 2A). Thus, this moderate destabilization is hypothesized to promote the release of cleaved RNA fragments, which then accelerates RNase H turnover and enhances antisense activity. However, SNA substitution at the central gap region (specifically G6–G10) reduced antisense activity *in vitro* (Fig. 4A) and decreased the efficiency of RNA cleavage by RNase H (Fig. 4C). In addition, the substitution resulted in a shift in the cleavage fragment length toward longer products. A previously reported crystal structure has indicated that the catalytic domain of RNase H recognizes a 7-nt segment of the DNA within a DNA/RNA duplex [47]. The site of RNA cleavage is spatially determined in relation to the DNA binding site: it is positioned diagonally opposite the third phosphate from the 3′ end of the DNA recognition sequence. Consequently, on the RNA strand, cleavage occurs at the fifth phosphodiester bond from the 5′ end of the binding region [47]. The chemical modifications that perturb the helical structure within this recognition footprint can lead to a shift in the binding register of RNase H, which leads to a change in the cleavage site and, in some cases, a reduction in cleavage efficiency [45]. Therefore, observed shifts in the cleavage fragment length and reduction in the efficiency of SNA-substituted Gapmers are likely attributed to a spatial misalignment. Specifically, SNA-induced structural perturbations alter the binding register of RNase H and prevent the catalytic residues from achieving the precise geometric orientation required to hydrolyze the target RNA phosphodiester bond. Consequently, maximizing the antisense potency of the modification is contingent upon balancing their thermodynamic advantages and precise positional selection to ensure productive RNase H engagement.

It is hypothesized that these effects are the result of conformational mismatch and structural perturbation arising from the chemical structural difference derived from the acyclic backbone. This structural difference is likely to prevent the heteroduplex from adopting the exact geometry required for the optimal binding of RNase H. As RNase H recognizes a specific footprint of the DNA/RNA heteroduplex to determine its cleavage site, these structural deviations can shift the binding position of RNase H or reduce binding affinity. Conversely, highly active Gapmers, such as 5w2, 5w3, and G1, appear to circumvent this interference. Based on the reported structure of the human RNase H1/RNA–DNA hybrid complex, 5w2, 5w3, and G1 are not expected to directly contact hRNase H1 [47]. Therefore, SNA substitution at these sites may moderately decrease *T*_m_ without perturbing the principal RNase H1 contact sites. Thus, appropriate positioning of SNA may help exploit a turnover-enhancing effect while avoiding substantial interference with RNase H-mediated cleavage. This strategy facilitates the concurrent optimization of safety and potency.

In conclusion, the present study has established SNA and L-*a*TNA as versatile chemical options for the rational modification of Gapmer therapeutics. By deviating from the conventional ribose-based scaffold and introducing an acyclic backbone, we achieved a superior safety profile than observed with standard 2′-OMe modifications. The present findings have identified the prevention of P54nrb mislocalization as the fundamental mechanism through which these acyclic analogs mitigate hybridization-independent cytotoxicity. Furthermore, we demonstrated that the strategic replacement of these modifications can exploit the modulation of *T*_m_ to enhance enzymatic turnover and achieve high potency while concurrently reducing toxicity. This study provides a robust molecular framework to support the design of next-generation antisense oligonucleotides that can surpass the traditional trade-offs between safety and efficacy.

## Supplementary Material

ugag036_Supplemental_File

## Data Availability

All data needed to evaluate the conclusions are provided in the main text and/or the supplementary materials.
