# Supplementary material for: Acyclic artificial nucleic acid substitution expands the safe design space of gapmer ASOs by preventing P54nrb mislocalization
Source: NAR Mol Med. 2026 Jul 6;3(3):ugag036. doi: 10.1093/narmme/ugag036 (PMC13385997; doi:10.1093/narmme/ugag036)
Supplement: ugag036_Supplemental_File [file ugag036_supplemental_file.docx]

Supplemental information

**Acyclic Artificial Nucleic Acids Substitution Expands the Safe Design Space of Gapmer ASOs by Preventing P54nrb Mislocalization**

Jumpei Ariyoshi,^1^ Masaya Higuchi,^2^ Hiroyuki Oyama,^1^ Hiroyuki Asanuma,^2^ Yukiko Kamiya ^1,2,*^

1 *Laboratory of Bioanalytical Chemistry, Kobe Pharmaceutical University, 4-19-1, Motoyamakita-machi, Higashinada-ku, Kobe, 658-8558, Japan*,

2 *Department of Biomolecular Engineering, Graduate School of Engineering, Nagoya University, Furo-cho, Chikusa-ku, Nagoya, 464-8601, Japan,*

*Corresponding author:  [y-kamiya@kobepharma-u.ac.jp](mailto:y-kamiya@kobepharma-u.ac.jp)

Figure S1. SNA or L-*a*TNA substitution at the G2 position reduces Gapmer cytotoxicity in 3T3-L1 cells. Cytotoxicity of the indicated modified Gapmers was evaluated in 3T3-L1 cells using the MTS assay. Data are shown as mean ± SD (*n* = 3).

Alt text (Figure S1): Bar graphs showing MTS assay results for 3T3-L1 cells treated with *Sod1-, Pcsk9-, Srb1-*, and *Acsl1*-targeting Gapmers containing SNA or L-*a*TNA substitution at the G2 position.

Figure S2. Positional effects of SNA substitution on Gapmer cytotoxicity in 3T3-L1 cells. Cytotoxicity of 3T3-L1 cells treated with each Gap-*Cxcl12* bearing a single SNA substitution at each position were evaluated using the MTS assay. Data are shown as mean ± SD (*n* = 3).

Alt text (Figure S2): Bar graphs showing MTS assay results for 3T3-L1 cells treated with Gap-*Cxcl12* derivatives containing a single SNA substitution at each position, highlighting position-dependent effects on cytotoxicity.


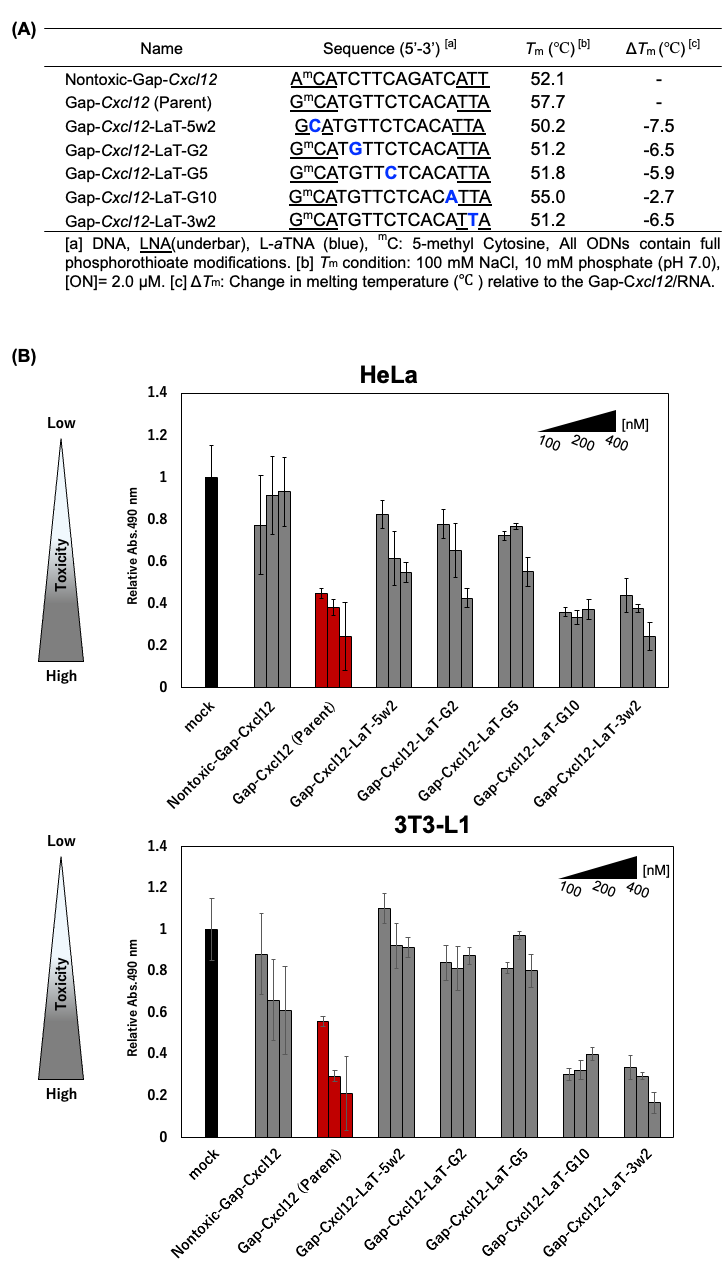


Figure S3. Positional effects of L-*a*TNA substitution on Gapmer cytotoxicity. (A) Sequences of the parental Gapmer (Gap-*Cxcl12*) and its L-*a*TNA-substituted Gapmers (e.g. Gap-*Cxcl12*-LaT-G2) and melting temperature (*T*_m_) of the Gapmer/complementary RNA duplex. (B) Cytotoxicity of HeLa and 3T3-L1 cells treated with each Gap-*Cxcl12* with a single L-*a*TNA substitution at each position, as determined by an MTS assay. Data are shown as mean ± SD (*n* = 3).

Alt text (Figure S3): Table listing Gap-*Cxcl12* sequences with single L-*a*TNA substitutions and corresponding melting temperatures, and a bar graph showing HeLa and 3T3-L1 cell viability (MTS assay) across Gap-*cxcl12*, a nontoxic control, and L-*a*TNA-substituted Gapmers at increasing concentrations.


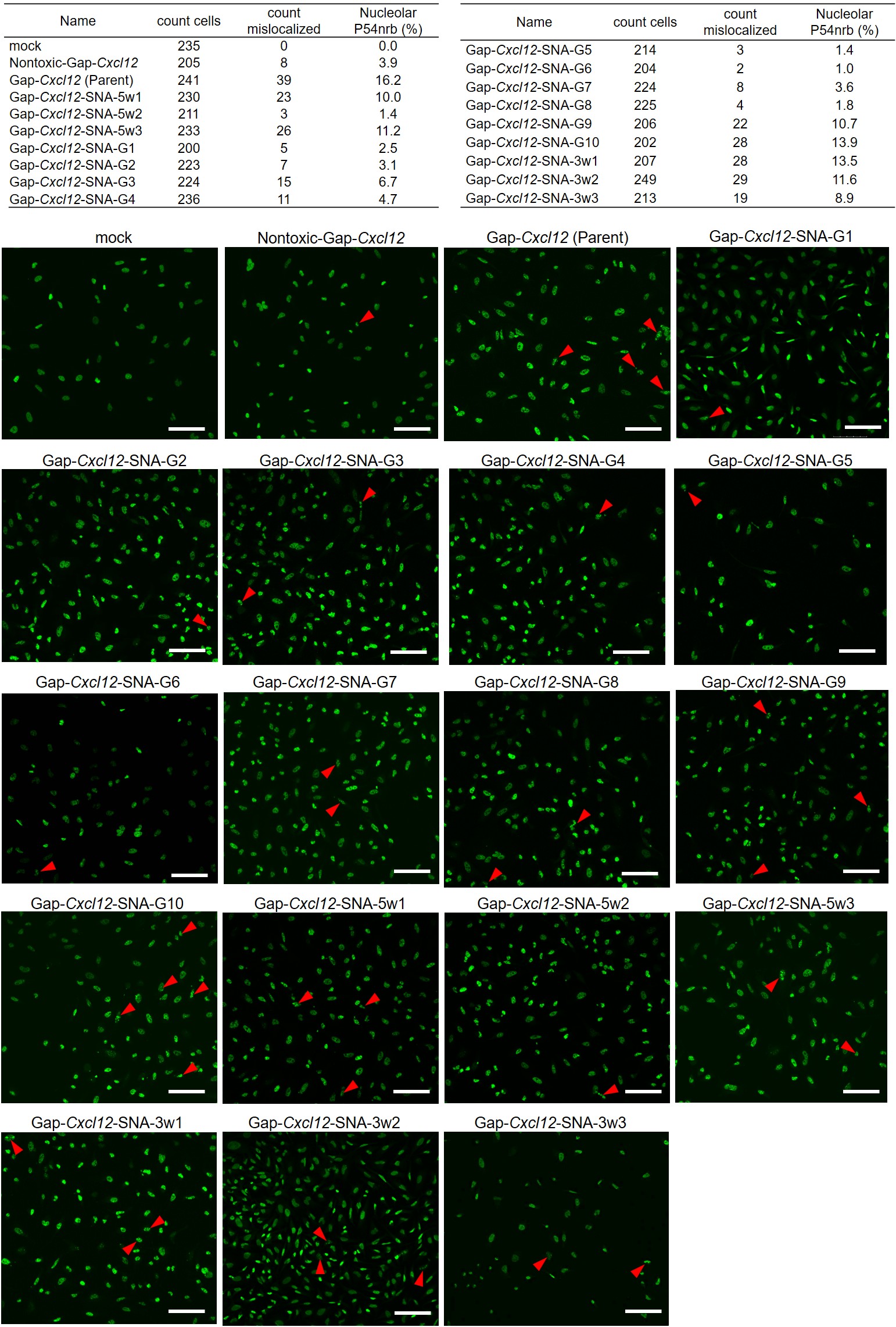


Figure S4. Immunofluorescence study of P54nrb in HeLa cells. HeLa cells were transfected with Gap-*Cxcl12* or SNA substituted Gap-*Cxcl12* for 24 h with 1,200 nM. Representative microscopic immunostaining images of P54nrb and percent of P54nrb mislocalization to nucleoli are shown each Gapmer. Scale bar, 100 µm.

Alt text (Figure S4): Grid of immunofluorescence images of HeLa cells after transfection with parent Gap-*cxcl12* or single SNA-substituted Gapmers (including wing and gap positions), showing nuclear P54nrb staining with nucleolar mislocalization marked by red arrowheads, plus tables summarizing cell counts and the percentage of cells with nucleolar P54nrb for each condition.


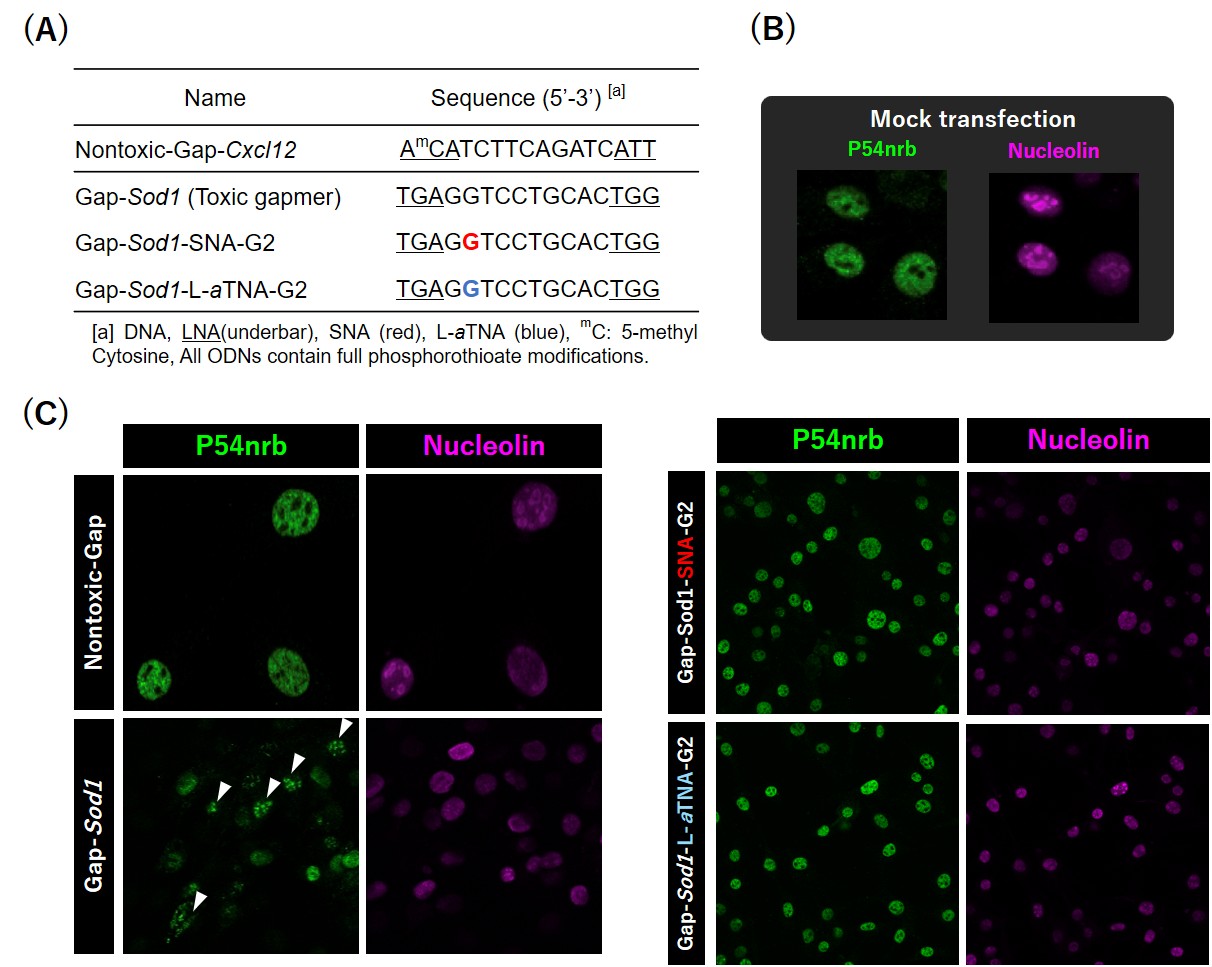


Figure S5. Localization of P54nrb in 3T3-L1 cells transfected with Gap-*Sod1*.

(A) Sequences of Gap-*Sod1* and its SNA or L-*a*TNA substitution at position G2. (B) Immunofluorescence staining of P54nrb (green) and nucleolin (magenta) in mock-treated 3T3-L1 cells. (C) Immunofluorescence staining of P54nrb (green) and nucleolin (magenta) in 3T3-L1 cells at 4 h after transfection with 1,200 nM Gap-*Sod1,* Gap-*Sod1*-SNA-G2*, or* Gap-*Sod1*-L-*a*TNA-G2.. White arrows indicate the mislocalization of P54nrb to the nucleolus.

Alt text (Figure S5): Sequence table and immunofluorescence images showing P54nrb and nucleolin localization in mock-treated and Gap-*Sod1*-transfected 3T3-L1 cells, with arrows marking nucleolar P54nrb mislocalization.

Figure S6. Additional serum biochemistry parameters following administration of SNA- or L-*a*TNA-substituted Gapmers *in vivo*. Male C57BL/6J mice (5 weeks old) were administered a single intravenous (*i.v.*) injection of PBS (mock), non-toxic Gap-*Cxcl12*, Gap-*Cxcl12*, or SNA- or L-*a*TNA-substituted Gap-*Cxcl12* at positions G2, G10, or 5w2 (Gap-*Cxcl12*-SNA-G2, -G10, -5w2, and Gap-*Cxcl12*-LaT-G2, -G10, -5w2) at a dose of 10 mg/kg. Blood samples were collected 96 h post-injection for biochemical analysis of total protein (TP), urea nitrogen (UN), creatinine (CRE), and alkaline phosphatase (ALP). Data are expressed as mean ± SD (n = 4 per group). Statistical analysis was performed using Welch’s t-test with Holm correction for multiple comparisons (each group vs. Gap-*Cxcl12*); no statistically significant differences were detected (all comparisons, ns).

Alt text (Figure S6): Mouse study schematic (*i.v.* dosing on day 0, sacrifice on day 4) and bar graphs of serum total protein, urea nitrogen, creatinine, and alkaline phosphatase at 96 h after dosing with Gap-*Cxcl12* or SNA/L-*a*TNA-substituted Gapmers, showing no significant differences versus Gap-*Cxcl12.*
